# Predicting inhibitory and activatory drug targets by chemically and genetically perturbed transcriptome signatures

**DOI:** 10.1038/s41598-017-18315-9

**Published:** 2018-01-09

**Authors:** Ryusuke Sawada, Michio Iwata, Yasuo Tabei, Haruka Yamato, Yoshihiro Yamanishi

**Affiliations:** 1 Division of System Cohort, Medical Institute of Bioregulation, Kyushu University, 3-1-1 Maidashi, Higashi-ku, Fukuoka, Fukuoka, 812-8582 Japan; 2 RIKEN Center for Advanced Intelligence Project, Nihonbashi 1-chome Mitsui Building, 15th floor, 1-4-1 Nihonbashi, Chuo-ku, Tokyo, 103-0027 Japan; 3 PRESTO, Japan Science and Technology Agency, Kawaguchi, Saitama, 332-0012 Japan

## Abstract

Genome-wide identification of all target proteins of drug candidate compounds is a challenging issue in drug discovery. Moreover, emerging phenotypic effects, including therapeutic and adverse effects, are heavily dependent on the inhibition or activation of target proteins. Here we propose a novel computational method for predicting inhibitory and activatory targets of drug candidate compounds. Specifically, we integrated chemically-induced and genetically-perturbed gene expression profiles in human cell lines, which avoided dependence on chemical structures of compounds or proteins. Predictive models for individual target proteins were simultaneously constructed by the joint learning algorithm based on transcriptomic changes in global patterns of gene expression profiles following chemical treatments, and following knock-down and over-expression of proteins. This method discriminates between inhibitory and activatory targets and enables accurate identification of therapeutic effects. Herein, we comprehensively predicted drug–target–disease association networks for 1,124 drugs, 829 target proteins, and 365 human diseases, and validated some of these predictions *in vitro*. The proposed method is expected to facilitate identification of new drug indications and potential adverse effects.

## Introduction

Genome-wide identification of all target proteins of drug candidate compounds is a challenging issue in drug discovery. Most drugs are small compounds that interact with target proteins to inhibit or activate their biological functions. However, drugs often interact with both primary targets and other proteins (off-targets). Thus, evaluations of possible pharmaceutical effects on single target proteins and multiple off-target proteins are required to improve the efficacy and safety of drug candidate compounds^1–3^ and to identify new indications for existing drugs (drug repositioning)^4,5^.

Numerous computational methods have been proposed for genome-wide drug target prediction using chemogenomic approaches based on compound chemical structures and protein sequences or structures^6–10^ and using phenotypic approaches based on drug side effects and similarities^11–14^. In addition, drug-induced gene expression profiles in human cell lines offer promise for predicting drug targets^15–19^. However, these methods depend heavily on the knowledge of ligands for target proteins, and fail to distinguish between inhibitory and activatory effects on target proteins.

Discrimination between inhibitory and activatory targets is crucial in many stages of drug development^20,21^. The related phenotypic effects (therapeutic and adverse effects) are heavily dependent on the inhibition or activation of target proteins. For example, drugs that activate dopamine receptors are used as Parkinson’s disease medications, whereas drugs that inhibit dopamine receptors are used as antipsychotic medications. In addition, target proteins remain unidentified for more than 60% of approved drugs, and only 50% of known drug–target interactions have annotations of inhibitory or activatory effects, according to our survey of existing chemical databases^22–28^.

Herein, we show that these problems can be addressed by integrating gene expression profiles following chemical induction and genetic perturbation. Accordingly, we postulated that, if a compound inhibits a certain protein, the ensuing gene expression profile can be correlated with that after gene knock-down of the corresponding protein. Likewise, if a compound activates a certain protein, the gene expression profile after chemical treatment of the compound may be correlated with that after over-expression of the protein. Based on this hypothesis, we developed novel methods that predict inhibitory and activatory targets of drug candidate compounds based on transcriptomic changes in global patterns of gene expression following chemical inhibition or induction and gene knock-down or over-expression. Finally, we confirmed the utility of these methods in analyses of pharmaceutical modes of action and in drug repositioning for a wide range of diseases.

## Results

### Overview of the proposed methods

**Figure 1:**
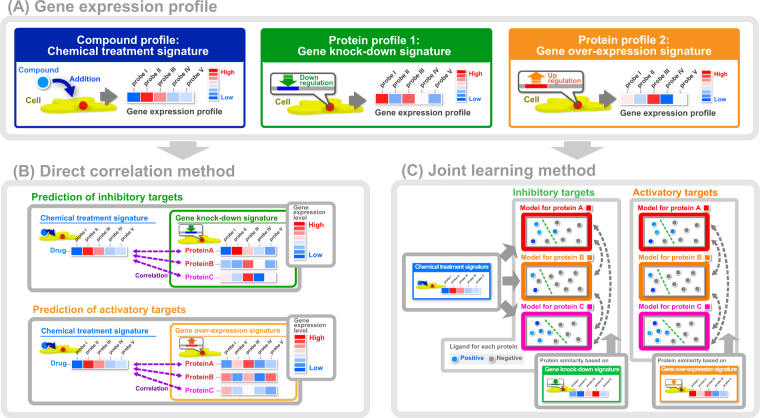
Data processing flow chart of the proposed method for predicting drug targets from transcriptome signatures of chemical and genetic perturbations; Panel (A) illustrates three types of gene expression profile for compounds and proteins. Compounds are represented by gene expression profiles after chemical treatment (chemical treatment signature). Proteins are represented by gene expression profiles after gene knock-down of the protein (gene knock-down signature), or by gene expression profiles after over-expression of the protein (gene over-expression signature). Panel (B) illustrates the direct correlation (DC) method. Correlation coefficients for inhibitory interaction pairs were calculated from chemical treatment and gene knock-down signatures. Correlation coefficients for activatory interaction pairs were calculated from chemical treatment and gene over-expression signatures. Panel (C) illustrates the joint learning (JL) method. Predictive models for individual target proteins were simultaneously learned by sharing protein similarities of gene knock-down and gene over-expression signatures.

Initially, we constructed gene expression profiles (signatures) for compounds and proteins by introducing three types of perturbations into human cell lines. These included chemical treatment, gene knock-down, and gene over-expression (Fig. 1A). 20,122 compounds were represented by chemical treatment signatures, whereas 4,331 proteins were represented by gene knock-down signatures and 2,946 proteins were represented by gene over-expression signatures.

In the direct correlation (DC) method (Fig. 1B), we calculated correlation coefficients between the chemical treatment and gene knock-down signatures, and between the chemical treatment and gene over-expression signatures for each of compound–protein pairs. Highly correlated compound–protein pairs were considered interacting pairs.

Figure 1C shows the joint learning (JL) method, in which predictive models are constructed for individual target proteins and are simultaneously learned to accommodate limited ligand information for target proteins. Models for inhibitory and activatory targets were then learned by sharing gene knock-down and over-expression similarities, respectively.

### Correlation of chemical and genetic perturbations

We investigated distributions of correlation coefficients of known compound–protein interaction pairs and non-interacting compound–protein pairs. In these computations, known interaction pairs tended to have higher correlation coefficients than the other pairs among both inhibition (p-value < 10^−20^) and activation interactions (p-value < 0.0237), indicating that chemical treatment with an inhibitor or activator is transcriptionally correlated with knock-down or over-expression of corresponding target protein. These results support the validity of the DC method. However, the correlation of chemical and genetic perturbations for activatory interactions is lower than that for inhibitory interactions. There is a possibility that the activation of a protein leads to reduction of its expression via negative feedback mechanisms. The low correlation of chemical and genetic perturbations for activatory interactions may be explained by the negative feedback mechanisms.

**Figure 2:**
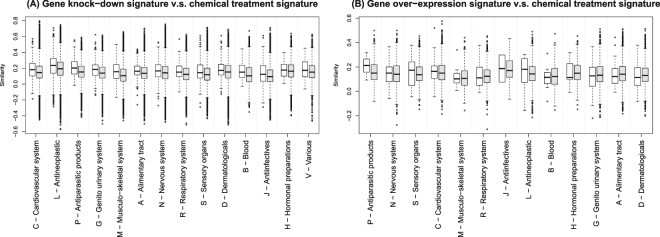
Distribution of correlation coefficients between chemical treatment signatures of inhibitors and gene knock-down signatures of the target proteins (**A**) and between chemical treatment signatures of activators and gene over-expression signatures of the target proteins (**B**). The corresponding box-plots are shown according to the first level of Anatomical Therapeutic Chemical (ATC) classification of drugs. White boxes indicate similarities of known interacting pairs, while gray boxes indicate similarities of the other pairs.

In experiments with a subset of compounds, we examined approved drugs for which Anatomical Therapeutic Chemical (ATC) classification groups have been assigned. Figure 2A shows distributions of correlation coefficients between chemical treatment and gene knock-down signatures, and Fig. 2B shows distributions of correlation coefficients between chemical treatment and gene over-expression signatures. Observed tendencies were dependent on ATC groups. For example, in the case of gene knock-down signatures, the tendencies were strong in Cardiovascular system and Antineoplastic groups, but were weak in Hormonal preparations and Antiinfective groups. In addition, the observed tendencies were strong in the inhibition, while the observed tendencies were relatively weak in the activation. These weak tendencies may reflect inclusion of non-interaction pairs that were previously unknown interaction pairs, and the ratio of previously unknown interaction pairs may differ between ATC groups, suggesting the presence of many unknown interaction pairs.

### Performance evaluation

We tested the ability of the proposed DC and JL methods to reconstruct inhibitory and activatory compound–protein interactions from gold standard data using cross-validation (CV) experiments (see the Methods section). We compared the proposed methods with the pairwise learning (PL) method that is widely used in chemogenomics (see Supplementary Information), and evaluated performance using receiver operating characteristic (ROC) and precision-recall (PR) curves. Results are summarized as areas under the ROC curves (AUC) and areas under PR curves (AUPR).

Tables S1 and S2 show the results of CV experiments using DC, PL, and JL methods, and AUC and AUPR scores of individual target proteins and their averages are presented. Because multiple cell lines were included for identical compounds and proteins, we applied cell-averaging and cell-concatenating operations (see the Methods section). The JL method gave higher AUC and AUPR scores than DC and PL methods, indicating that supervised learning with known compound–protein interactions is relevant. In the JL method, cell-averaging operations worked better than cell-concatenating operations in most cases. One explanation about the low performance of the DC method is that the number of known compound–protein interactions is very limited. Note that the DC method is an unsupervised approach, while the proposed JL method is a supervised approach. Because the DC method is unsupervised, its accuracy may be underestimated, reflecting previously unknown interactions. If potentially true compound-protein interactions were regarded as negative examples in the gold standard data, the accuracy scores would be low in the case of unsupervised approach. The DC method did not work in the case of activation, but the JL method improved the accuracy even in the case of activation.

**Figure 3:**
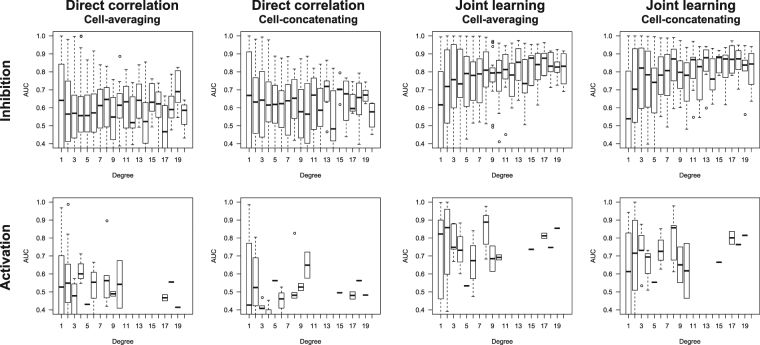
Distributions of AUC scores for individual target proteins; AUC scores are plotted against numbers of known ligands (degree) for each protein. Upper and lower rows indicate results for inhibition and activation benchmark datasets, respectively.

Figure 3 shows AUC scores based on numbers of known ligands for each protein for DC and JL methods using inhibition and activation benchmark datasets. AUC and AUPR scores for DC, PL, and JL methods are shown in Figures S3 and S4, respectively. AUC scores tended to be lower for lower degree values, whereas AUC scores tended to be higher with higher degree values. Hence, predictions are difficult when numbers of known ligand compounds in the learning set are small. Moreover, prediction accuracy for low degrees was better maintained with the JL method than with DC and PL methods.

**Figure 4:**
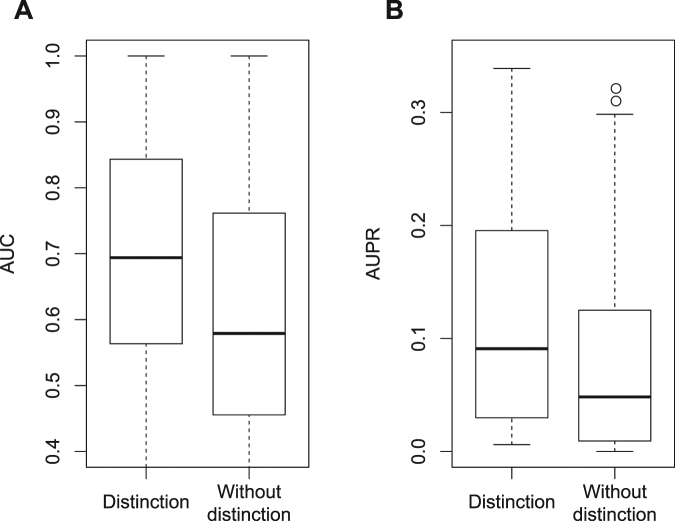
Performance evaluation of drug indication predictions. Panels A and B show AUC and AUPR scores for diseases, respectively, in the presence and absence of distinctions between inhibitory and activatory targets.

Furthermore, we evaluated performances of target-based drug indication predictions and sensitivities to differences between inhibitory and activatory targets based on known therapeutic targets for diseases (see the Methods section). Figure 4 shows AUC and AUPR scores for diseases in drug indication predictions with and without distinctions between inhibitory and activatory targets. Note that previous methods for predicting drug indications were performed without distinguishing between inhibition and activation. Accuracy was greater with distinction than without distinction. Hence, distinctions between inhibitory and activatory targets are crucial for appropriate evaluations of the therapeutic effects of candidate drug compounds on human diseases.

To investigate the sensitivity of the performance to the effect of the ratio of negative compounds against positive compounds in the training set, we generated a balanced training set by sampling such that the ratio of negative compounds against positive compounds is 3 (positive vs negative = 1:3) for each target protein, and performed additional cross-validation experiments. We compared the cross-validation results between the balanced training set and the original training set (positive vs negative = 1:all), where in the both cases we used the same test set and evaluated all compounds in the test set. Table S3 in Supplementary Information shows the corresponding AUC and AUPR scores, and Figure S6 in Supplementary Information shows the box-plots of the corresponding AUC and AUPR scores by degrees. It was observed that the AUC and AUPR scores of the balanced training set were lower than those of the original training set. We used all negative compounds in the training set for constructing a predictive model in this study.

### Large-scale prediction of drug–target–disease networks

Herein, we applied the proposed methods to drug repositioning and predicted new indications of 1,124 drugs (registered in Japan, USA, and Europe) for 365 human diseases. Initially, we estimated target proteins for these drugs using the JL method, for which predictive models were learned using all gold standard data as training data. We focused on the top 5% predictions, which produced 42,174 inhibitory drug–protein interaction pairs among 760 drugs and 755 proteins and 4,141 activatory drug–protein interaction pairs among 622 drugs and 74 proteins.

**Figure 5:**
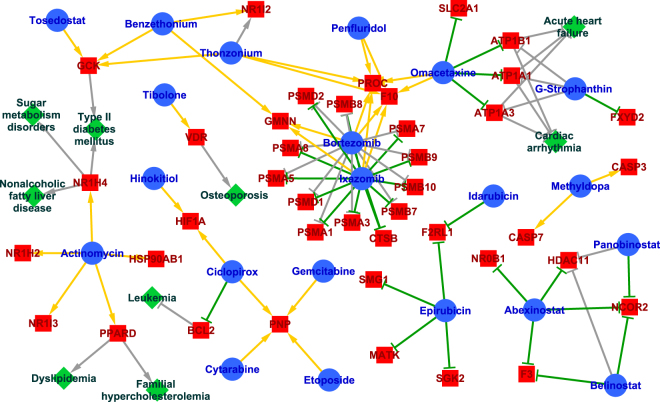
A small portion of the drug–protein–disease network predicted by the joint learning method. Blue circles indicate drugs, red rectangles indicate proteins, and green diamonds indicate diseases. Orange arrows indicate newly predicted activatory interactions and green T-shapes indicate newly predicted inhibitory interactions. Gray arrows indicate known activatory associations and gray T-shapes indicate known inhibitory associations.

Subsequently, we comprehensively predicted novel drug indications with target profiles comprising known targets and newly predicted targets, based on known therapeutic targets of diseases (see Methods section). We confirmed the validity of several prediction results using independent resources that were absent from the learning data. For example, the antifungal drug ciclopirox was predicted to inhibit B-cell lymphoma 2 (BCL-2) as a treatment for leukemia. BCL-2 is a recently identified therapeutic target that is highly expressed in various cancer cell types, and its inhibition has antiproliferative effects^29^, as indicated by antileukemia effects of ciclopirox^30^. Tibolone is prescribed to ease menopausal symptoms, but was predicted to activate the vitamin D receptor (VDR). Because vitamin D deficiency decreases bone density and increases the risk of osteoporosis^31^, Tibolone has been predicted to have efficacy as a treatment for osteoporosis, and corresponding therapeutic effects were reported^32^. Figure 5 shows a small portion of the resulting drug–target–disease association network, which provides mechanistic insights into predicted drug indications. Networks involving the confirmed examples and other predicted pairs with high prediction scores for inhibitory and activatory interactions were generated by using Cytoscape^33^.

### Experimental validation in *in vitro* assays

We focused on retinoic acid receptor α (RAR *α*) as a target protein. RAR *α* is a nuclear receptor that is involved in signal transduction for cellular maturation and differentiation^34^, and is required for estrogen-related cell profiles^35^. Inhibition of RAR *α* induced apoptosis in breast cancer cells^36^ and RAR *α* silencing inhibited cancer cell proliferation^37^. Thus, the inhibition of RAR *α* may lead to therapeutic effects in estrogen-related cancers such as breast and ovarian cancers.

We focused on sulfamethoxypyridazine, prenylamine lactate, and dienestrol that were top 3 compounds predicted to inhibit RAR *α*. We tested the activities of the three compounds in cellular assays (see the Methods section and Supplementary Information for more details). We were able to confirm the activity of dienestrol, but unable to confirm the activities of sulfamethoxypyridazine and prenylamine lactate.

**Figure 6:**
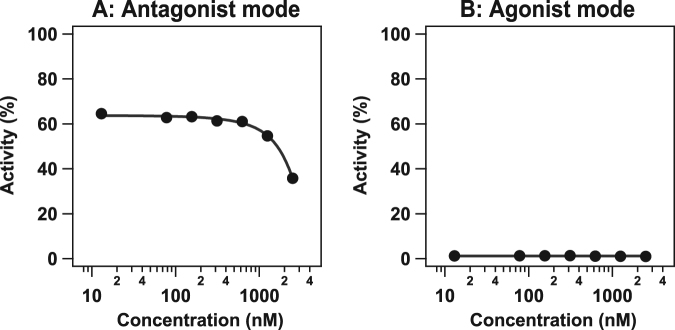
Dose response curve of dienestrol (solid line) in the RAR *α* assay in the antagonist and agonist modes. The horizontal axis shows the log concentration of dienestrol. The vertical axis shows percentage dienestrol activity. Circles represent data points from triplicate experiments.

Figure 6 shows the dose response curve of dienestrol in the antagonist mode. In these experiments, dienestrol antagonized the RAR *α* with an IC_50_ of 2.75 *μ*M. Moreover, percentage activity decreased from approximately 65% to 35%. No agonistic effects were observed. These experimental results validate the prediction that dienestrol inhibits signal transduction via RAR *α*. Thus, the anti-psychotic drug dienestrol may be useful for the treatment of estrogen-related breast and ovarian cancers.

## Discussion

In this study, we propose novel methods for predicting inhibitory and activatory targets of drug compounds on a genome-wide scale. The present methods are novel integrations of chemically and genetically perturbed transcriptome data, and can be used to discriminate between inhibitory and activatory targets. Furthermore, simultaneous predictions for multiple target proteins improved the accuracy for proteins with limited ligand information. Finally, we demonstrated the utility of the proposed methods for predictions of drug targets and indications. We suggest that the proposed methods will facilitate the understanding of modes of action of candidate drug compounds.

Phenotype-based high-throughput screening (PHTS) can be used to identify drug candidate compounds that lead to desired phenotypes^38^. However, the underlying molecular mechanisms of hit compounds identified by PHTS remain unknown, and further investigations are required to determine target proteins with desired phenotype associations^39,40^. To this end, the present methods can be used to relate phenotypic effects of hit compounds with corresponding target proteins. Drug repositioning may also be a promising application of the proposed method, because although various computational methods for systematic drug repositioning have been developed using molecular data^16,41–50^, most of these are purely predictive and lack biological relevance. In contrast, the present method can indicate comprehensive drug–target–disease networks in which inhibitory and activatory targets are distinguished for drugs and diseases.

Another promising application of the proposed method may be in the prediction of adverse drug effects^13,51–53^. For example, drugs that inhibit dopamine receptors should not be prescribed for Parkinson’s disease, because dopamine agonists are medications for Parkinson’s disease. Similarly, drugs that activate dopamine receptors should not be prescribed for psychotic patients, because some anti-psychotics drugs are inhibitors of dopamine receptors. Accordingly, the present method facilitates evaluations of risk in clinical applications.

As a result of investigating our hypothesis, we showed that inhibitors (resp. activators) were correlated with inhibitory targets (resp. activatory targets) in terms of gene expression patterns, but these correlations were sometimes weak. We also showed that the weak correlations could be overcome to some extent by simultaneous prediction with a machine learning technique. However, there remains much room for the improvement of the proposed method. For example, the identification of features predictive towards the labels and the improvement of cell-averaging/cell-concatenating operations are important tasks. We would like to tackle these problems as important future works.

## Methods

### Chemically-induced and genetically-perturbed transcriptome

Gene expression profiles from the Library of Integrated Network-based Cellular Signatures (LINCS) project were obtained from the Broad Institute’s website (http://download.lincs-cloud.org/)^54^, and the effects of chemical treatments, gene knock-down, and gene over-expression were compared. In this study, we used gene expression profiles of chemical treatments to represent drug features. Subsequently, we analyzed gene expression profiles following gene knock-down to represent features of inhibitory target proteins, and gene expression profiles following gene over-expression to represent features of activated target proteins. Gene expression levels were measured using flow cytometry, and test samples were prepared using 384-well plates. LINCS provided 978 landmark genes (L1000 genes). We used the expression of 978 landmark genes as the gene expression signatures in this study.

We prepared three types of gene expression profiles, including drug candidate compounds, inhibitory target proteins, and activatory target proteins (Fig. 1A). We selected 663,572 chemical treatment signatures (“trt_cp”), 448,737 gene knock-down profiles (“trt_sh”), 86,267 gene over-expression profiles (“trt_oe”), and 81,342 control profiles (“ctl_”). We then normalized gene expression profile values to corresponding control profiles and calculated z-scores. Compounds with chemical treatment signatures and proteins encoded by the genes that were identified in gene knock-down and over-expression signatures were converted to InChIKey (http://www.iupac.org/home/publications/e-resources/inchi.html) and KEGG GENE IDs^26^, respectively. We obtained a total of 114,642 chemical treatment signatures including 20,122 compounds and 71 cell lines, 37,558 gene knock-down signatures including 4,331 proteins and 20 cell lines, and 19,859 gene over-expression signatures including 2,946 proteins and 10 cell lines.

Gene expression profiles for single drugs or genes were generated using multiple cell lines. Thus, we used cell-averaging and cell-concatenating operations. For cell-averaging operations, all cell line profiles were averaged, and in cell-concatenating operations, all cell line profiles were concatenated into a single profile.

### Inhibitory and activatory compound–protein interactions

Inhibitory and activatory interactions of compound–protein pairs, including drug–protein pairs, were obtained from the seven public databases ChEMBL^22^, MATADOR^23^, DrugBank^24^, Psychoactive Drug Screening Program Ki (PDSP-Ki)^25^, KEGG DRUG^26^, BindingDB^27^ and Therapeutic Target Database^28^.

For ChEMBL, we selected only compound–protein interaction pairs that were clearly denoted as active interactions or had a binding affinity less than 30 *μ*M. We used the following criteria to select inhibition or activation pairs from the interactome database. We selected the drug–protein pair with defined inhibition or activation interactions (definition field includes the”inhibitor”,”activator”,”antagonist” or”agonist”). In addition, we selected interaction pairs with inhibitory binding affinity units (IC50 and Ki) as inhibition pairs. We selected compound–protein pairs with defined inhibitory or activatory interactions using the following definition fields: inhibitor, activator, antagonist, and agonist, and selected interaction pairs with inhibitory binding affinity data. In total, 10,031 compound–protein inhibitory interactions (2,445 compounds and 769 proteins) and 432 compound–protein activatory interactions (350 compounds and 77 proteins) were obtained. The compound–protein interactions consisted of compounds and proteins from chemical treatment signatures, gene knock-down signatures, and gene over-expression signatures were used as gold standard data in CV experiments to evaluate the performance of drug target estimation.

### Direct correlation (DC) method

To predict inhibitory and activatory interactions, correlation coefficients of chemical treatment signatures and gene knock-down signatures, and correlation coefficients of chemical treatment signatures and gene over-expression signatures were calculated, respectively. Highly correlated compound–protein pairs were considered candidate interaction pairs, and the corresponding correlation coefficients were used as prediction scores.

### Joint learning (JL) method

Individual predictive models were constructed for multiple target proteins, and the models simultaneous learning was achieved by sharing protein similarities based on gene knock-down and over-expression profiles.

In these computations, we predicted whether or not a given compound *X*_*i*_(*i* = 1,2, … , *N*) would inhibit or activate the *m*-th target proteins (*m* = 1, 2, … , *M*). We then constructed statistical models defined as ${f}_{m}^{inh}({X}_{i})={{\bf{w}}}_{m}^{inh\,{\rm{T}}}\varphi ({X}_{i})$ and ${f}_{m}^{act}({X}_{i})={{\bf{w}}}_{m}^{act\,{\rm{T}}}\varphi ({X}_{i})$, where compound *X*_*i*_ is represented by the chemical treatment signature $\varphi ({X}_{i})$, ${{\bf{w}}}_{m}^{inh}$ is a weight vector for inhibiting the *m*-th target protein, and ${{\bf{w}}}_{m}^{act}$ is a weight vector for activating the *m*-th target protein. To accommodate compound actions that are poorly characterized, we learned individual predictive models ${f}_{1}^{inh},{f}_{2}^{inh},\ldots \,,{f}_{M}^{inh}$, by sharing known gene knock-down perturbations across *M* target proteins, and learned ${f}_{1}^{act},{f}_{2}^{act},\ldots \,,{f}_{M}^{act}$, respectively, by sharing known gene over-expression perturbations across *M* target proteins. Details of the algorithm are described in the Supplementary Information.

### Performance evaluation protocol

To perform CV experiments, all compounds in the gold standard compound–target interaction datasets were split into five subsets for use as test data and the other subsets were used as training data. Target proteins of test compounds were then predicted using a predictive model that was constructed with training data. Finally, prediction accuracy was evaluated using prediction scores of all test compounds.

The number of positive samples is much smaller than that of negative samples in the gold standard datasets. In this study, there are 10,031 positives and 1,870,174 negatives for inhibition and 432 positives and 26,518 negatives for activation. In general, the imbalanced sets produce low AUPR values. In practice the number of negative examples is much larger than that of positive examples, thus it is important to simulate such practical situations in the cross-validation experiments. We evaluated the accuracy for individual proteins and calculated the average over the proteins. Many proteins have few ligands (e.g., only one or two drugs inhibiting/activating the proteins), thus it is very difficult to learn on such a small number of positive examples. Thus, the resulting AUPR scores tend to be low.

### Drug indication predictions

Drug indications (applicable diseases) were predicted from target profiles of drugs, including primary targets, off targets, and target profiles of diseases. For each drug–disease pair, we identified inhibitory and activatory target proteins that appeared in the drug target profile and in the disease target profile. Drugs were then linked to diseases when at least one common target protein could be distinguished in terms of inhibition and activation. We repeated this procedure for all drug–disease pairs and extended previous methods^50^ by accommodating differences between inhibition and activation. Details are described in the Supplementary Information.

### *In vitro* assays

The effects of compounds against human retinoic acid receptor alpha (RAR *α*) were determined using Mammalian one-hybrid type GAL4-Reporter Gene Assays, which were performed by Phenex Pharmaceuticals AG using HEK293 cells (DSMZ ACC 305). Assays were conducted in two modes, agonist and antagonist, where the compound was tested at 9 concentrations with 2 vehicle controls in quadruplicate. In the antagonist mode, lower levels of luminescence were observed for higher concentrations of antagonists, whereas in the agonist mode, higher levels of luminescence were observed for higher concentrations of agonists. More details of the experimental procedures are presented in the Supplementary Information.

## Electronic supplementary material

Supplementary Information
