# Supplementary material for: Predicting inhibitory and activatory drug targets by chemically and genetically perturbed transcriptome signatures
Source: Sci Rep. 2018 Jan 9;8:156. doi: 10.1038/s41598-017-18315-9 (PMC5760621; doi:10.1038/s41598-017-18315-9)
Supplement: Supplementary file 1 — Supplementary Information [file 41598_2017_18315_MOESM1_ESM.pdf]

SUPPLEMENTARY INFORMATION

Predicting inhibitory and activatory drug targets by chemically and genetically perturbed transcriptome signatures

Ryusuke Sawada[1], Michio Iwata[1], Yasuo Tabei[2], Haruka Yamato[1], & Yoshihiro Yamanishi[1,3]*

[1]*Division of System Cohort, Medical Institute of Bioregulation, Kyushu University, 3-1-1 Maidashi, Higashi-ku, Fukuoka, Fukuoka 812-8582, Japan.*

[2]*RIKEN Center for Advanced Intelligence Project, Nihonbashi 1-chome Mitsui Building, 15th floor, 1-4-1 Nihonbashi, Chuo-ku, Tokyo 103-0027, Japan.*

[3]*PRESTO, Japan Science and Technology Agency, Kawaguchi, Saitama 332-0012, Japan.*

*Correspondence to Yoshihiro Yamanishi [yamanishi@bioreg.kyushu-u.ac.jp]

Methods

Pairwise learning (PL) method As a comparison method to the proposed method, we use the pairwise learning method which is one of the widely-used prediction methods in chemogenomics and pharmacogenomics fields. Figure S1 shows an illustration of the pairwise learning method. In this method, we prepared a feature vector for each drug–protein pair by concatenating a chemical treatment signature and a gene knock-down signature, or by concatenating a chemical treatment signature and a gene over-expression signature. Each drug–protein pair is assigned a label repre-

senting interaction or non-interaction. Then, we learned a predictive model from drug–protein pair feature vectors. For the prediction of inhibition pairs, we use chemical treatment signatures and gene knock-down signatures. In addition, for the prediction of activation pairs, we use chemical treatment signatures and gene over-expression signatures. The prediction algorithm is described detailed below.

Drug–protein interaction prediction can be formulated by supervised classification of drug–protein pairs. A pair made up of drug X and protein Y is represented by (X, Y). Each drug–protein pair (X, Y) is represented by a feature vector as $\Phi(X, Y)$. We use a function, $f(X, Y) = \mathbf{w}^{\mathrm{T}}\Phi(X, Y)$, to predict whether the drug–protein pair (X, Y) is an interacting pair or not. The weight vector $\mathbf{w}$ is optimized on the basis of a learning set with label information. The learning set consists of drug–protein pairs (X_i, Y_j) $(i = 1, 2, \ldots, N; j = 1, 2, \ldots, M)$, where the drug–protein pairs are known to be interacting pairs (positive pairs) or not (negative pairs). N is the number of drugs and M is the number of proteins in the learning set.

The profile of drug X is represented by a p-dimensional vector as follows: $X = (x_1, x_2, \ldots, x_p)^{\mathrm{T}}$, where p is the number of landmark genes (978 in the case of the cell-averaging operation). In the same manner, the profile of protein Y is represented by a q-dimensional vector as follows: $Y = (y_1, y_2, \ldots, y_q)^{\mathrm{T}}$, where q is the number of landmark genes (978 in the case of the cell-averaging operation).

We represent each drug–protein pair as a feature vector concatenating of X and Y as follows:

$$\Phi(X, Y) = (x_1, \ldots, x_p, y_1, \ldots, y_q)^{\mathrm{T}}, \tag{1}$$

where $\Phi(X, Y)$ is a $(p + q)$-dimensional feature vector.

We consider a learning set of drug–protein pairs and interaction labels $(\Phi(X_i, Y_j), a_{ij})$, $a_{ij} \in \{+1, -1\}$ $(i = 1, 2, \ldots, N; j = 1, 2, \ldots, M)$, where $a_{ij} = 1$ if drug i interacts with protein j and $a_{ij} = -1$ if drug i does not interact with protein j. The weight vector $\mathbf{w}$ of the linear logistic regression is learned with L_1 regularization as follows:

$$\min_{\mathbf{w}} ||\mathbf{w}||_1 + \lambda \sum_{i=1}^{N} \sum_{j=1}^{M} \log(1 + \exp(-a_{ij}\mathbf{w}^{\mathrm{T}}\Phi(X_i, Y_j))), \tag{2}$$

where $|| \cdot ||_1$ is L_1 norm (the sum of absolute values) and λ is a regularization parameter to control the sparsity penalty. To handle the over-fitting problem, we introduced L_1 regularization in the learning process. L_1 regularization has an effect that makes the weights of uninformative features zeros without a loss of classification accuracy. Recently, efficient learning algorithms of linear models for high-dimensional feature vectors have been developed and commonly used in many practical applications [1]. The dependent variable is an interaction label: yes (+1) or no (-1). We used all possible drug–protein pairs excluding positive pairs as negative pairs to train the model.

In the pairwise learning method, samples correspond to drug–protein pairs, features correspond to concatenated signatures (chemical treatment signatures and gene knock-down/over-expression signatures), and labels correspond to interactions (+1 for interaction, -1 for non-interaction).

Joint learning (JL) method Here, the details of the algorithm of the joint learning method are described. For easier readability, f^{inh} and f^{act} in the main manuscript are described as f below.

In order to overcome the scarcity of pre-knowledge concerning relationships between compound–target pairs, we propose to jointly learn individual predictive models $f_1, f_2, \ldots, f_M$, sharing information across M target proteins.

We attempt to jointly estimate all the weight vectors $\mathbf{w}_1, \mathbf{w}_2, \ldots, \mathbf{w}_M$ in the models by minimizing the logistic loss as follows:

$$R(\mathbf{W}) = \sum_{j=1}^{M} \sum_{i=1}^{N} \log(1 + \exp(-a_{i,j} \mathbf{w}_j^{\mathrm{T}} \Phi(X_i))). \tag{3}$$

We introduce a regularization term $\Omega(\mathbf{W})$ to the loss function in order to enhance the generalization properties. Thus, the optimization problem is written as follows:

$$\min_{\mathbf{W}} R(\mathbf{W}) + \Omega(\mathbf{W}). \tag{4}$$

Here we introduce two kinds of regularization terms. First, we use a standard ridge regularization term to avoid the over-fitting problem, which is defined as

$$\Omega_r := \frac{1}{2}Tr(\mathbf{W}\mathbf{W}^{\mathrm{T}}). \tag{5}$$

Second, we design another regularization term reflecting the similarities among target proteins. In this study we evaluate the similarity of gene knock-down signatures for target proteins using Pearson's correlation coefficient in the prediction of inhibitory interactions, and the similarity of gene over-expression signatures for target proteins using Pearson's correlation coefficient in the prediction of activatory interactions. We construct an $M \times M$ similarity matrix S for target proteins in which each element $S_{i,j}$ is a similarity score between the i-th and the j-th target proteins. Then, we introduce the following regularization term:

$$\begin{aligned} \Omega_s(\mathbf{W}) &:= \frac{1}{4}\sum_{j=1}^{M}\sum_{k=1}^{M} S_{j,k}\left\|\frac{\mathbf{w}_j}{\sqrt{K_{j,j}}} - \frac{\mathbf{w}_k}{\sqrt{K_{k,k}}}\right\| && (6)\\ &= \frac{1}{2}Tr(\mathbf{W}L_s\mathbf{W}^{\mathrm{T}}), && (7)\end{aligned}$$

where $\|\cdot\|$ is the Euclidean norm, K is a diagonal matrix defined as $K_{j,j} := \sum_{k=1}^{M} S_{j,k}$, and L_s is a symmetric normalized Laplacian defined as $K^{-1/2}(K-S)K^{-1/2}$. The regularization term $\Omega_s(\mathbf{W})$ has the effect of making the weight vectors $\mathbf{w}_j$ and $\mathbf{w}_k$ close to each other if $S_{j,k}$ is high. Finally, we introduce the following regularization term in the optimization problem eq. (4):

$$\Omega(\mathbf{W}) := \lambda_s\Omega_s(\mathbf{W}) + \lambda_r\Omega_r(\mathbf{W}), \tag{8}$$

where $\lambda_s \geq 0$ and $\lambda_r \geq 0$ are hyper-parameters to control the strength of the regularization

terms Ω_s and Ω_r, respectively. If the λ_s value is large, the protein similarities are taken into account in the learning process. On the other hand, if the λ_s value is small, the protein similarities are not taken into account in the learning process. The details explanations on the rationale of the joint learning can be found in machine learning papers [2,3].

In the joint learning method, samples correspond to drugs, features correspond to chemical treatment signatures, and labels correspond to interactions (+1 for interaction with the m-th target protein, -1 for non-interaction with the m-th target protein).

In general, the AUPR value tends to be low when the number of positive examples is very few [4]. In this study the number of known ligands (corresponding to positive examples) with information on inhibitors/activators (corresponding to labels) is few for most proteins. It implies that the problem addressed in this paper is very challenging. Note that the AUPR values of the proposed method are much higher than those of random inference. An imbalanced training set (very large number of negatives) is a problem for the supervised learning method. If positive examples and negative examples are equally handled with the same weight, the supervised learning method is likely to become a negative-biased predictor. To avoid such a problem, in the learning process based on the training set, we put a small weight (i.e., the number of positive examples / the number of negative examples) on the loss functions for negative examples in equation (3) in supplementary information. This is how we avoided constructing a negative-biased predictive model.

Drug indication predictions Inhibitory and activatory associations of disease–protein pairs were manually collected from published data. Inhibitory disease–protein associations were those in

which inhibition of the protein provides favorable disease treatment. Conversely, activatory disease–protein associations were those for which activation of the protein provides favorable disease treatment. Finally, 1,052 inhibitory disease–protein associations were identified among 223 diseases and 185 proteins and 41 activatory disease–protein associations were identified among 25 diseases and 21 proteins. Specific diseases are represented by target profiles in which inhibitory or activatory target proteins are coded as 1 or 0, respectively. We put disease–protein association data on the following website: http://www.bioreg.kyushu-u.ac.jp/labo/systemcohort/inh-act/

***in vitro* assay** An *in vitro* cellular mammalian one-hybrid type GAL4-Reporter Gene Assay was performed by Phenex Pharmaceuticals AG. We tested three drugs, sulfamethoxypyridazine (Sigma-Aldrich, #46858), prenylamine lactate (LGC Standards, #LGCFOR1000.00), dienestrol (Santa Cruz, #SC-239717) against the human retinoic acid receptor alpha (RARα).

HEK293 cells (DSMZ ACC 305) were transformed with the plasmids used in the Phenex GAL4 assay system. The plasmids were derivatives of the Stratagene M2H reporter plasmid; the reporter plasmids were a modified pFR-Luc, containing a synthetic promoter with two tandem repeats of the yeast GAL4-binding sites that control the expression of the *Photinus pyralis* luciferase gene, and pCMV-BD. For the binding assay, a plasmid constitutively expressing a PGC1α fragment was also used to obtain reasonable assay quality. A second reporter (pRL-CMV), with *Renilla reniformis* luciferase driven by a constitutive promoter, was also used to improve the accuracy of the experiments.

The *Photinus* luciferase activity value was divided by the *Renilla* luciferase activity value

and multiplied by 1,000 (the resulting value is denoted as "RENnorm"). RENnorm was calculated for each well and used for the calculation of percent activity values as follows:

$$(\text{Percent activity}) = \frac{(\text{RENnorm}) - \tilde{C}_v}{\tilde{C}_r - \tilde{C}_v} \times 100,$$

where $\tilde{C}_v$ and $\tilde{C}_r$ represent the median values for DMSO vehicle (C_v; negative control) and a reference compound (C_r; positive control), respectively, calculated per assay plate. The activity in the negative control wells (DMSO vehicle only) equals to 0%, and that in the positive control wells (saturating concentration of a reference compound) equals to 100%. The reference compounds were TTNPB (CAS NO. 71441-28-6) and Ro41-5253 (CAS NO. 144092-31-9) for the agonist and antagonist modes, respectively.

The Igor Pro software (WaveMetrics, Lake Oswego, OR) was used to calculate the dose response curves and EC50/IC50 values using a four-parameter logistic function.

Results

Correlation of chemical and genetic perturbations according to compound–protein interactions We calculated the correlation coefficients between chemical treatment signatures and gene knock-down signatures. Figure S2A shows the p-values of the difference between interacting pairs and non-interacting pairs. We calculated the correlation coefficients between chemical treatment signatures and gene over-expression signatures. Figure S2B shows the p-values of the difference between interacting pairs and non-interacting pairs.

Performance evaluation Tables S1 and S2 show the results of the cross-validation (CV) experiments for the direct correlation, pairwise learning and joint learning methods using cell-averaging and cell-concatenating operations. We calculated the AUC and AUPR scores in two different ways: using global evaluation and using local evaluation. In the global evaluation, we calculated the AUC and AUPR scores for all drug–protein pairs at one time; whereas in the local evaluation, we calculated the AUC and AUPR scores of individual target proteins and took their averages over all target proteins.

Relationship between accuracy and number of known ligands of each protein Figure S3 shows the AUC scores based on the degrees (the number of positive examples for each protein) for direct correlation, pairwise learning and joint learning methods using inhibition and activation benchmark datasets. In addition, the AUPR scores are shown in Figure S4.

Performance comparison between learning with protein similarities and learning without protein similarities Figure S5 shows the scatter-plots of the AUC scores for individual proteins by the joint learning method with protein similarities against those without protein similarities. It was observed that the accuracy was improved for proteins with low degrees (proteins with few ligands), but the accuracy was not improved for proteins with high degrees (proteins with many ligands). The results suggest that the joint learning is effective when predictive models are learned on a small number of positive examples in the training set, and the local learning is encouraged when predictive models are learned on a large number of positive examples.

Large-scale prediction of drug–target–disease networks We focused on the analysis of top 5% predictions of drug–protein pairs. We put the newly predicted drug–protein interaction pairs on the following website: http://www.bioreg.kyushu-u.ac.jp/labo/systemcohort/inh-act/

Reference

1. Fan, R.-E., Chang, K.-W., Hsieh, C.-J., Wang, X.-R. & Lin, C.-J. Liblinear: A library for large linear classification. *Journal of Machine Learning Research* **9**, 1871–1874 (2008).

2. Bickel, S., Bogojeska, J., Lengauer, T. & Scheffer, T. Multi-task learning for hiv therapy screening. *Proceedings of the 25th International Conference on Machine Learning* 56–63 (2009).

3. Nori, N., Kashima, H., Yamashita, K., Ikai, H. & Imanaka, Y. Simultaneous modeling of multiple diseases for mortality prediction in acute hospital care. *Proceedings of the 21th ACM SIGKDD International Conference on Knowledge Discovery and Data Mining* 855–864 (2015).

4. Davis, J. & Goadrich, M. The relationship between precision-recall and roc curves. In *Proceedings of the 23rd International Conference on Machine learning*, 233–240 (ACM, 2006).

Table S1: Performance evaluation for three methods by CV experiments for drug target predictions with local evaluation.

Method	Random	Direct correlation		Pairwise learning		Joint learning	
		Cell-averaging	Cell-concatenating	Cell-averaging	Cell-concatenating	Cell-averaging	Cell-concatenating
Inhibition							
AUC	0.500	0.527	0.610	0.659	-	**0.714**	0.702
AUPR	0.005	0.013	0.020	0.027	-	0.060	**0.073**
Top10 ratio	0.045	0.091	0.122	0.216	-	0.235	**0.259**
Activation							
AUC	0.500	0.527	0.485	0.488	0.472	**0.746**	0.661
AUPR	0.016	0.030	0.023	0.028	0.022	**0.106**	0.094
Top10 ratio	0.122	0.104	0.104	0.091	0.091	**0.312**	0.247

”-” means that it was not computationally feasible, because the size of training data was too large to learn the predictive model.

Table S2: Performance evaluation for three methods by CV experiments for drug target predictions with global evaluation.

Method	Random	Direct correlation		Pairwise learning		Joint learning	
		Cell-averaging	Cell-concatenating	Cell-averaging	Cell-concatenating	Cell-averaging	Cell-concatenating
Inhibition							
AUC	0.500	0.571	0.561	**0.860**	-	0.796	0.849
AUPR	0.053	0.008	0.007	0.055	-	0.039	**0.105**
Activation							
AUC	0.500	0.526	0.538	0.781	0.797	0.785	**0.799**
AUPR	0.016	0.017	0.018	0.098	0.107	0.121	**0.209**

”-” means that it was not computationally feasible, because the size of training data was too large to learn the predictive model.

Table S3: Performance evaluation for joint learning method with cell-averaging by CV experiments in using the original training set and the balanced training set (positive vs negative = 1:3) for drug target predictions with local evaluation.

Training set	Original	Balanced
Inhibition		
AUC	0.714	0.644
AUPR	0.060	0.046
Top10 ratio	0.235	0.202
Activation		
AUC	0.746	0.637
AUPR	0.106	0.072
Top10 ratio	0.312	0.221

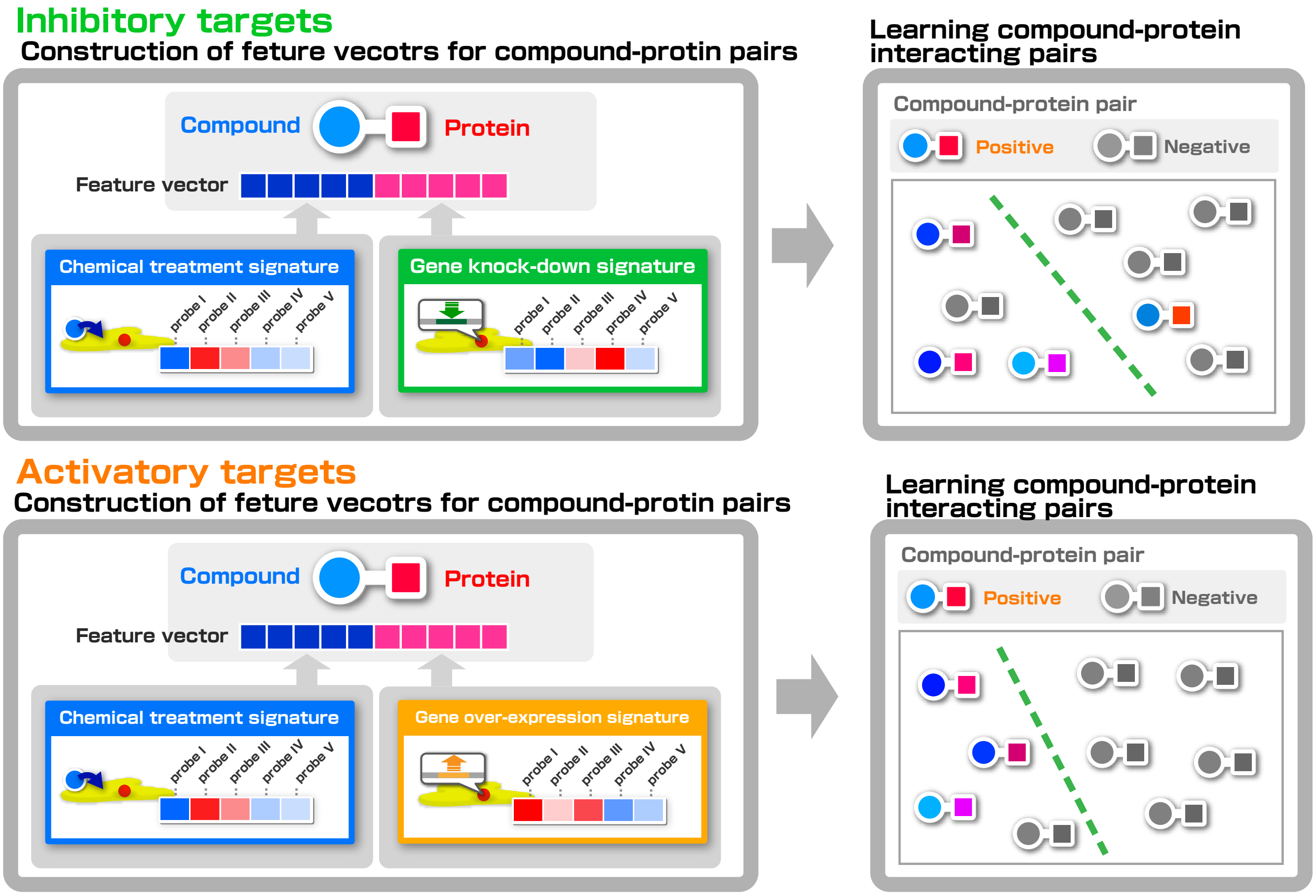

Figure S1: Basic idea of the pairwise learning method. A feature vector for each drug–protein pair is prepared by concatenating the drug profile and the protein profile. For the prediction of inhibitory interactions, chemical treatment signatures and gene knock-down signatures are used. For the prediction of activatory interactions, chemical treatment signatures and gene over-expression signatures are used.

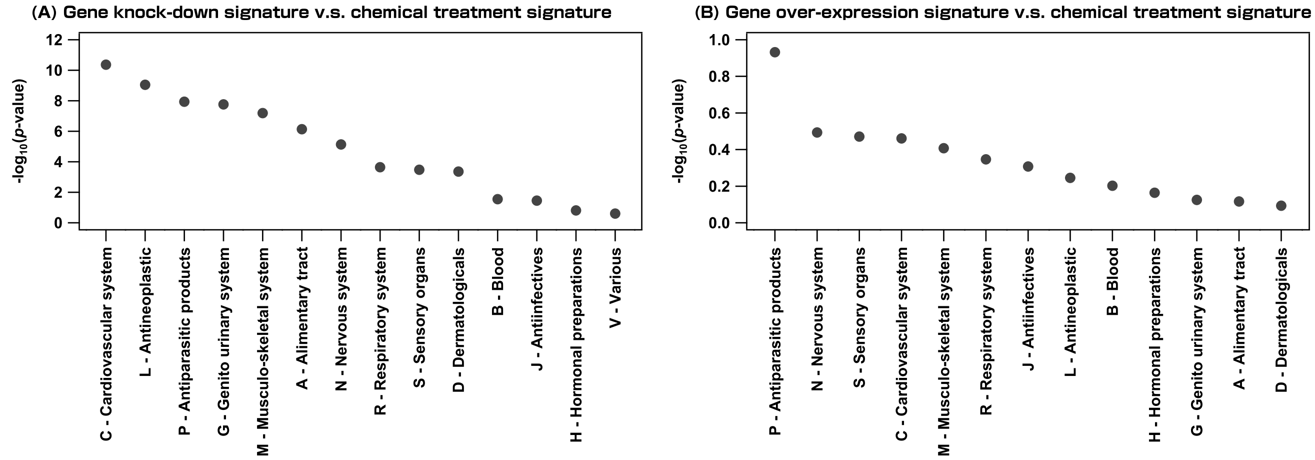

Figure S2: Differing correlation coefficients between compound–protein interaction pairs and non-interaction pairs. Panel (A) shows differences between chemical treatment signatures of inhibitors and gene knock-down signatures of target proteins and panel (B) shows differences between chemical treatment signatures of activators and gene over-expression signatures of target proteins. Corresponding box plots are shown according to the first level of Anatomical Therapeutic Chemical (ATC) drug classifications.

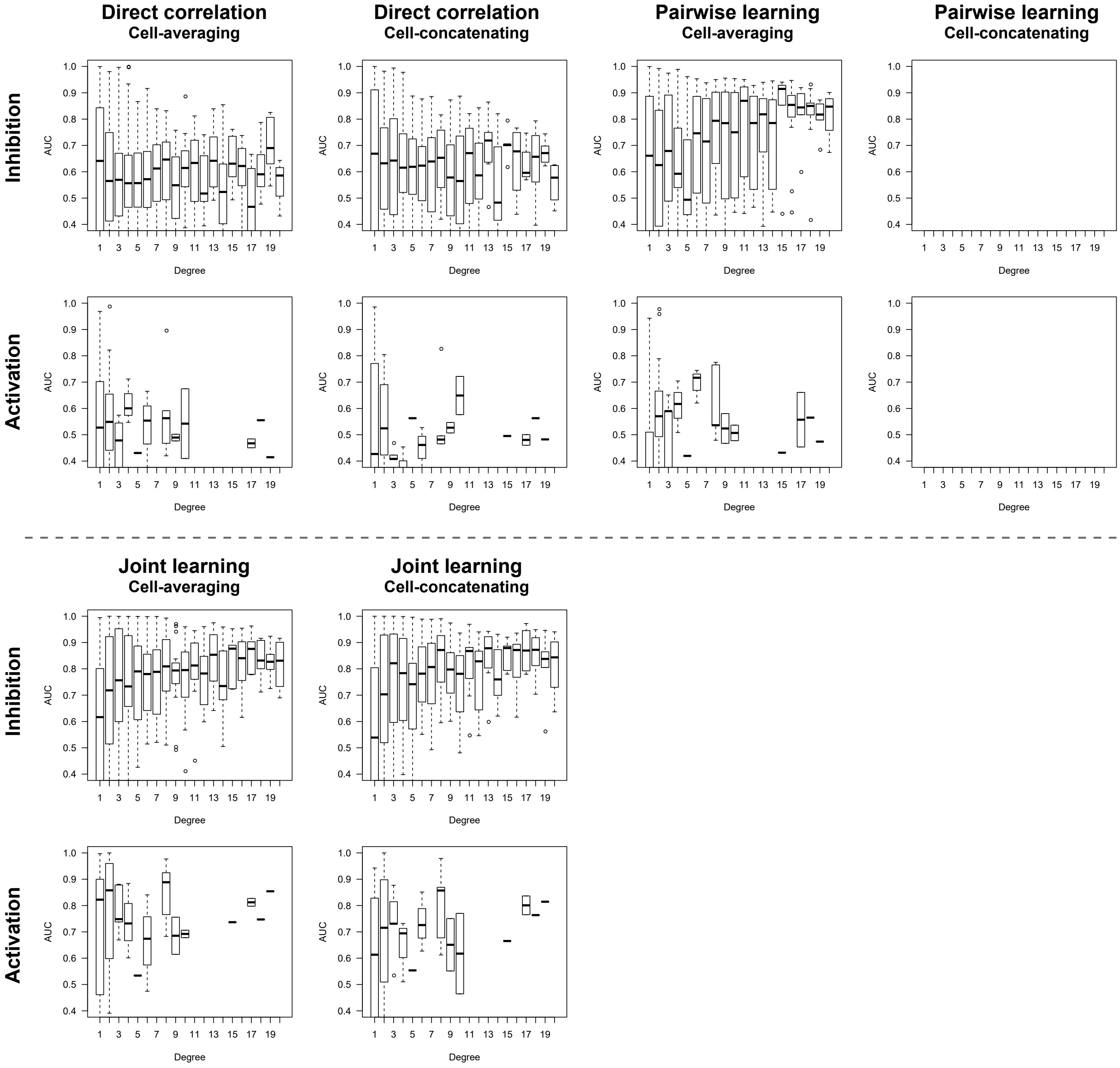

Figure S3: Index-plots of AUC scores from CV experiments. AUC scores are plotted by the degree which is the number of positive examples for each protein. Upper and lower rows indicate the results of CV experiments with inhibition data and activation data, respectively.

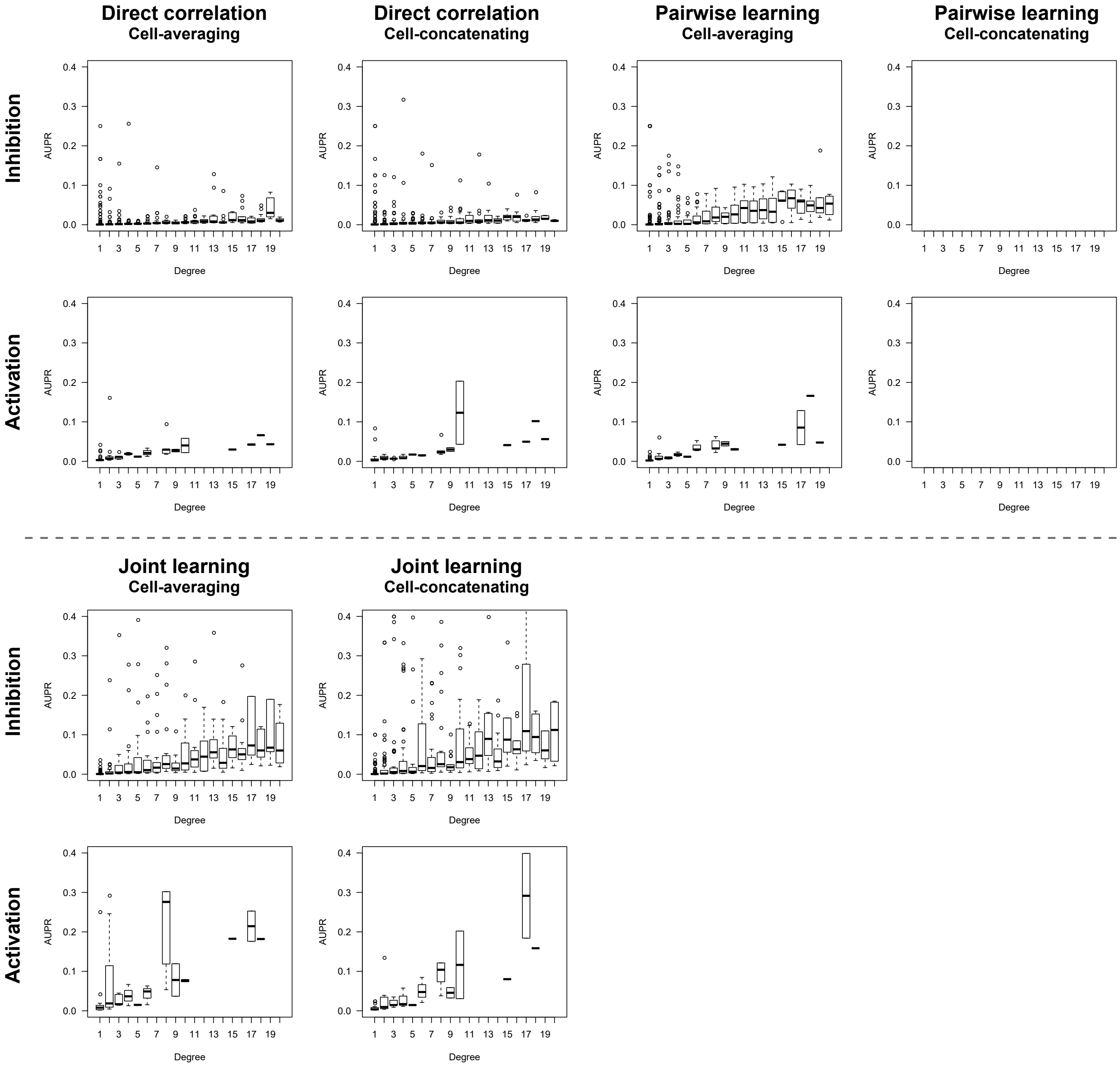

Figure S4: Index-plots of AUPR scores from CV experiments. AUPR scores are plotted by the degree which is the number of positive examples for each protein. Upper and lower rows indicate the results of CV experiments with inhibition data and activation data, respectively.

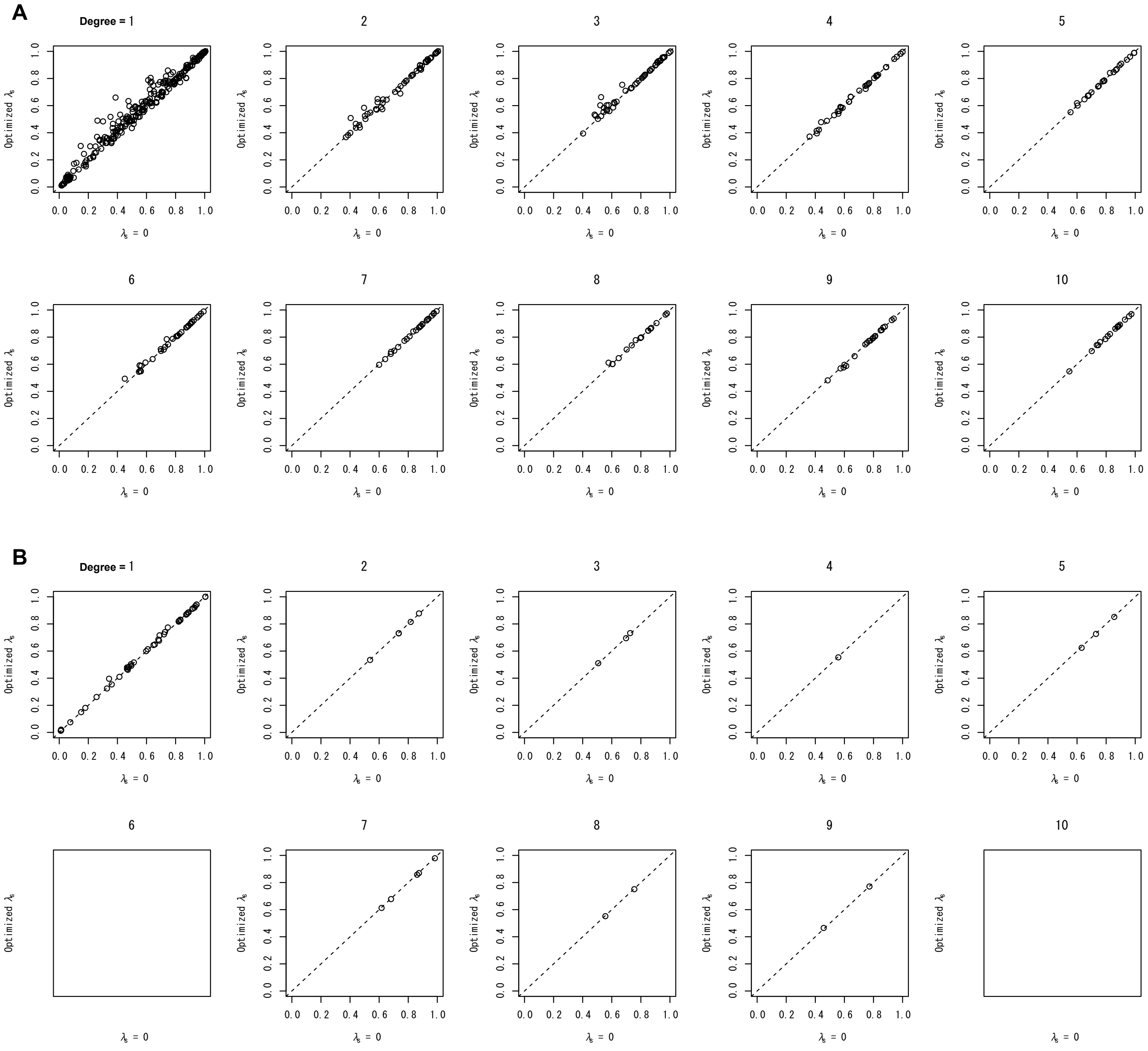

Figure S5: Comparison of the AUC scores between the joint learning method with protein similarities (optimized λ_s) and the joint learning method without protein similarities ($\lambda_s = 0$): CV experiment for inhibition data (A) and activation data (B).

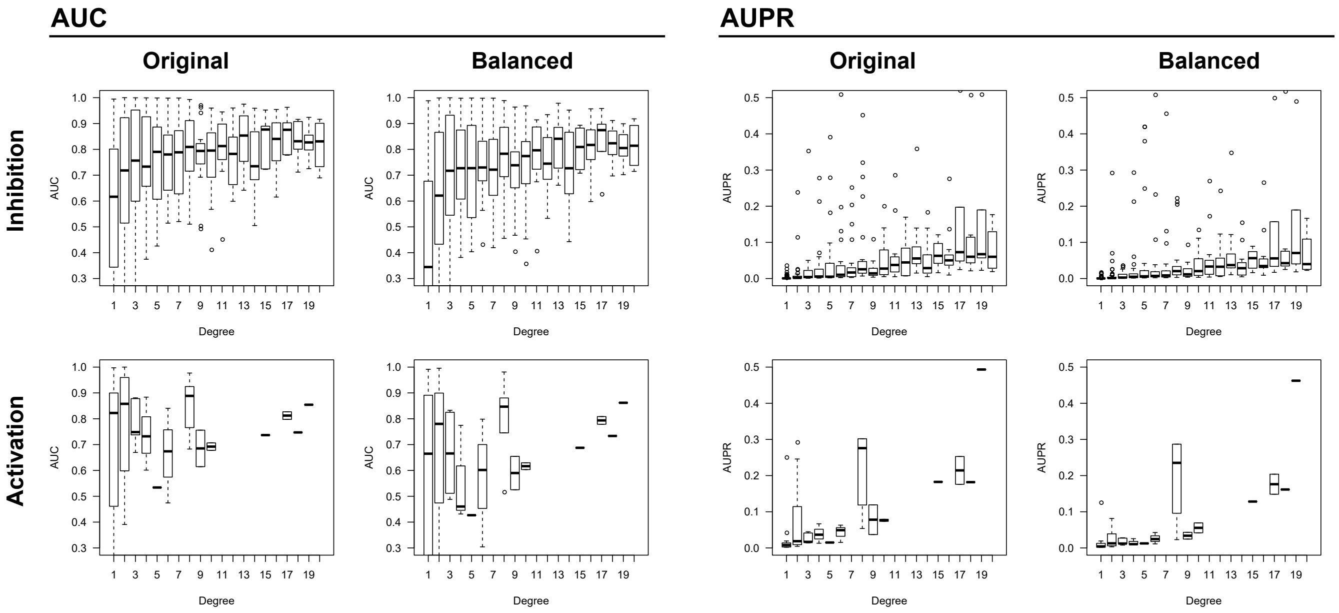

Figure S6: Index-plots of AUC and AUPR scores for joint learning method with cell-averaging from CV experiments in using the original training set and the balanced training set (positive vs negative = 1:3). AUPR scores are plotted by the degree which is the number of positive examples for each protein. Upper and lower rows indicate the results of CV experiments with inhibition data and activation data, respectively.
